# “Like an animal”: the well-being of women living in restricted housing units

**DOI:** 10.1186/s40352-023-00215-y

**Published:** 2023-03-08

**Authors:** Lindsay R. Smith, Sydney Ingel, Danielle S. Rudes

**Affiliations:** 1 George Mason University, Fairfax, USA; 2 Sam Houston State University, Huntsville, USA

**Keywords:** Prisons, Women, Wellness, Restricted housing units, United Nations

There are close to 100,000 people serving time in solitary confinement units in American prisons and jails each day (Department of Justice (2016)). Three times that number are subjected to a solitary confinement stay of 15 days or more every year (Beck, 2015), despite the United Nations declaring solitary confinement should be limited to sentences of 15 days or less for prison residents (Gannon, 2019; United Nations General Assembly, 2015). The United Nations also suggests that the use of solitary confinement for women is not appropriate (United Nations General Assembly, 2015) in line with research showing that solitary confinement has a disproportionate impact on people of color, youth, and women (Digard et al., 2018). Plus, women who are incarcerated tend to have higher rates of serious mental health disorders (SMHDs) compared to men who are incarcerated (LaChance, 2018). Unfortunately, there is a pattern of residents with mental health disorders being: (1) charged with prison rule violations more frequently, (2) subjected to solitary confinement more often, (3) and required to serve longer sentences in solitary confinement (Houser & Belenko, 2015; Wright et al., 2007). However, women are oftentimes more likely to be sentenced to solitary confinement for institutional misconducts, usually minor and nonviolent in nature, such as, talking back and refusing orders, as compared to men (Aranda-Hughes et al., 2021; Shaylor, 1998). In this way, women tend to receive more punitive in-prison sentences for less serious violations than men (LaChance, 2018) and this is especially true for women with mental health disorders or co-occurring disorders (Houser & Welsh, 2014). Furthermore, transgender women are also more likely to be placed in solitary confinement for their perceived protection from the rest of the resident population whether asked for by the resident or decided without their consent (Andasheva, 2016). Sometimes, this means that transgender women spend almost their entire prison sentence in solitary confinement, simply because they identify as transgender, a population that is highly marginalized and stigmatized, putting them at increased risk of victimization while incarcerated. Overall, solitary confinement is overused—arguably for everyone—but particularly for women, yet their experiences within it are underexplored.

## Literature review

The United States (U.S.) is the world’s leader in incarceration with over 650 individuals incarcerated per 100,000 people (Widra & Herring, 2021). The mass incarceration problem does not just affect men though, who make up the larger proportion of prison populations, women are also significantly impacted. In fact, the United States incarcerates 30% of the world’s women (Widra & Herring, 2021). Due to expanded law enforcement efforts, stiffer drug sentencing laws, and unaddressed reentry barriers, the number of women entangled in the criminal legal system has dramatically risen since the 1980s (The Sentencing Project, 2020). During this time, the number of women who became incarcerated increased by more than 700% (The Sentencing Project, 2020). As a result, women make up about 7% of the carceral population in America today (Carson, 2015). In 2019 alone, 222,455 women were incarcerated in prisons and jails in the U.S. (The Sentencing Project, 2020). These incarceration trends continue to result in prison overcrowding for residents of all genders, fiscal burdens for state and federal governments, and staff and resource shortages that make the carceral experience privy to strain (Martin et al., 2012; Pitts et al., 2014; The Sentencing Project, 2015).

With mass incarceration and subsequently under-resourced correctional institutions, the “pains of imprisonment” are just as present today as when Gresham Sykes laid them out in the 1950s (Sykes, 1958). In turn, residents may be more likely to act out in various ways to try to make their carceral experience better (Ingel et al., 2021b) and this behavior is highly likely to receive attention and punishment by correctional staff. Typically resulting in a solitary confinement sentence, women are then confined in a small cell with a sink, toilet, bed, mattress, limited light, minimal programming, few reading materials, and communication restrictions with friends and family (Ahalt et al., 2017; Cloud et al., 2015; Metcalf et al., 2013; Reiter, 2012). What often results is negative sensations, feelings, and cognitive functioning due to exacerbating mental health symptomatology and physical health disorders such as trouble sleeping, time distortion, intrusive thoughts, panic attacks, hallucinations and delusions, increased paranoia, hypersensitivity to light, reduced memory, feelings of dehumanization, and identity loss (Bersot & Arrigo, 2010; Metzner & Fellner, 2010; Reiter et al., 2020; Steinbuch, 2014; Western et al., 2021, Browne et al., 2011) In turn, Winters (2018) pointed out that women in solitary confinement “frequently engage in unhealthy relationships or alliances steeped in drama, verbally bully others, engage in physical aggression with officers, and create chaotic distractions” purported to provide a source of entertainment, garner some form of human touch, and/or reduce feelings of boredom or anger (p. 218). In a similar way, Martel’s (2001), Haney’s (2003), and Reiter et al. (2020) studies of women’s experiences in solitary confinement revealed that women would often go to great lengths to regain a sense of agency, such as through self-harm behavior or suicide attempts to try to regain control over their own bodies. Beyond hurting oneself, Strong et al. (2020) study on the physical health problems of residents who have experienced solitary confinement revealed the following medical issues occurred while staying in solitary confinement: skin irritations, weight fluctuation, un-treated/mistreated chronic conditions, and musculoskeletal pain. Often without the means to heal from physical and/or mental health problems, residents continue to live in pain throughout their solitary confinement stay, entire prison sentence, and even beyond institutional walls.

The underlying issues resulting in residents’ needs going unmet lies at the institutional level. Institutional constraints (e.g., staff and resource shortages) combined with institutional goals/priorities (e.g., safety, security, and control of residents) often take precedence over residents’ rights (Ingel et al., 2021a; Rudes et al., 2021). In fact, several Departments of Corrections (DOCs) across the United States do not guarantee the rights of residents but rather suggest that policies should be interpreted with flexibility for the purposes of carrying out DOC goals. Additionally, prison administrators operate their prisons with minimal court and federal oversight (Alderstein, 2001; Schlanger & Shay, 2008) meaning the day-to-day operations of prisons are largely left to the discretion of prison staff in charge of implementing amorphous policies from prison administrators (Foudray, C., Smith, L., McPherson, M., & Rudes, D. (2021). *Code grey: how correctional middle managers manipulate policy into practice for the betterment of the prison experience*. George Mason University. Unpublished manuscript). Although, the Federal Prison Oversight Act was introduced to the U.S. Congress in late September of 2022 which would require inspections of all facilities overseen by the Bureau of Prisons (BOP) as well as investigations of residents’ complaints regarding their wellbeing (Federal Prison Oversight Act, 2022). Thus, if the federal government of the U.S. has little control or even influence over prison operations currently, it is highly unlikely that prisons consider, much less adhere to, international standards regarding the treatment of individuals who are incarcerated. For instance, the United Nations recommends that correctional officers who are men should only access women’s units when entering alongside correctional officers who are women (Human Rights Committee, 2006 as cited in Cerneka, 2017). However, U.S. correctional officers that are men continue to be allowed to carry out job duties in women’s units, including cell extractions, strip searches, and being present while women use the bathroom/shower (Cerneka, 2017). These staff actions may trigger post-traumatic stress disorder (PTSD) symptoms related to several women’s trauma histories, including sexual and interpersonal violence (Arrigo & Bullock, 2008). Not having the resources to adequately address residents’ needs is one problem, but continuing to uphold institutional goals over policies written into law or guidelines offered at the international level meant to maintain the well-being of residents is problematic at best and inhumane at worst.

According to Nelson Mandela, “It is said that no one truly knows a nation until one has been inside its jails. A nation should not be judged by how it treats its highest citizens, but its lowest ones” (Mandela, 1994, p. 23). To ensure prison residents’ wellness globally, the General Assembly of the United Nations—of which the U.S. is a member—has adopted several sets of international standards regarding the treatment of incarcerated individuals. These include the: (1) *Basic Principles for the Treatment of Prisoners* (United Nations General Assembly, 1990), (2) *United Nations Rules for the Treatment of Women Prisoners and Non-custodial Measures for Women Offenders* (also known as the *Bangkok Rules*; United Nations General Assembly, 2010), and (3) *United Nations Standard Minimum Rules for the Treatment of Prisoners* (also known as the *Nelson Mandela Rules*; United Nations General Assembly, 2015). Collectively, these three documents of international standards set out what is generally accepted as good practices in the treatment of individuals who are incarcerated and the management of institutions in which they are housed. The focus is on basic standards that ensure the humane and dignified treatment of individuals while they are incarcerated, placing particular emphasis on successful rehabilitation and reentry. In addition, there are guidelines within the *Nelson Mandela Rules* regarding solitary confinement stays as well, in that indefinite solitary confinement or prolonged solitary confinement stays should not be allowed but ultimately solitary confinement should be used as a last resort (United Nations General Assembly, 2015). Furthermore, the rules insist that solitary confinement should not be used for residents with mental or physical health disorders if doing so means exacerbating those issues (United Nations General Assembly, 2015). Although these standards set forth by the General Assembly of the United Nations are not binding or enforceable, member nations are theoretically supposed to consider and implement them, and not doing so is grounds for criticism by other member nations and examination by sociological researchers.

### Current study

There has been no systematic attempt to investigate how closely U.S. prisons follow standards set forth by the United Nations, nor have there been studies examining potential consequences to incarcerated individuals such as poor wellness when prisons do not abide by them. Additionally, understanding the experiences of women who are incarcerated, an understudied population within U.S. prisons, offers a deeper examination of residents’ well-being that is not well understood currently as compared to men who are incarcerated. Furthermore, exploring women’s perspectives while living in solitary confinement provides a more nuanced view of those most marginalized and neglected in women’s prisons, and those likely with more needs. Therefore, using one U.S. women’s prison as a case study, we qualitatively examine semi-structured interviews with women residents about their wellness. Predicated on our semi-grounded theoretical approach, we highlight the experiences of women residents when United Nations standards are not adhered to during stays in solitary confinement. The women we interviewed were all housed in solitary confinement, otherwise known as the prison’s restricted housing units (RHUs), which are characterized by: (1) removal from the general prison population; (2) placement in a locked cell, whether alone or with another individual, and (3) inability to leave the cell for the majority of the day, typically 22 h or more (U.S. Department of Justice, 2016). Individuals can be placed in or sentenced to an RHU when they are perceived to pose a threat to themselves or others (e.g., high-ranking member of a gang, escape risk), they are perceived to need protection (e.g., perceived threat of victimization, celebrity figure), or as punishment for an infraction or misconduct (e.g., talking back, refusing orders). Thus, the group of women we interviewed are amongst the most likely to be negatively affected by non-adherence to the basic standards set forth by the United Nations for how incarcerated individuals should be treated and delving into their experiences is even more important for the foreseeable implications that research may have on improving women’s well-being in prison, but especially RHUs.

## Methodology

Researchers conducted a multi-year qualitative interview study at a large state correctional agency on the East Coast between 2017 to 2019. In both 2018 and 2019, researchers visited the same women’s prison. This prison was chosen because it is the only medium/maximum women’s prison in the state containing multiple RHUs and specialized RHUs (i.e., secure housing for individuals with mental health conditions and/or behavioral issues). Researchers manually confirmed that no resident was interviewed twice across the two trips using recruitment lists of residents from both years.

Although the overall research team consisted of 26 researchers, only about 10 researchers collected data during each trip. Data collection trips lasted two days with researchers noting observations of the units and conducting interviews with both RHU residents and RHU staff. Interviews and observations took place between 6 A.M. to 10 P.M. The first half-day of each trip to the institution was spent on participant recruitment. To recruit participants, the researchers approached each cell and introduced the project. After explaining that participation in an interview with us was voluntary, each resident was asked if they would like to participate. Residents were only deemed ineligible if they: (1) did not speak English^1^; (2) did not have the mental faculties to give consent; (3) were actively violent; (4) were asleep or not in their cell at time of recruitment, or (5) refused to participate. Residents who met eligibility criteria and agreed to participate had their names added to a hand-written recruitment list (agreement rate: about 90%). This list was then used to have residents pulled out of their cells for interviews, which ensured that correctional staff were not choosing interviewees for researchers. Although there was an initial agreement rate of about 90%, not all of those women were interviewed. This was due to reasons such as: (1) correctional staff deemed the resident too much of a risk to be interviewed, (2) residents left the RHU before they could be interviewed, or (3) the resident changed their mind about participating. Sometimes women residents changed their mind about participating because the interview co-occurred with some other activity (e.g., shower, programming) and we were unable to interview them at a more convenient time. In other cases, women decided the strip search they would have to endure upon leaving their cell outweighed their desire to interview with us.

In total, interviews were conducted with 44 women at one women’s prison. Interviews took place in locations such as visiting rooms, psychiatric cages,^2^ the law library, or in a supervisor’s office. To safeguard participant confidentiality, but also maintain the safety of the research team, interview locations were visible (but not audible) to staff via eye or camera. Interviews typically lasted 45 to 60 min each using an informal, semi-structured interview process. A semi-grounded theory approach was used for data collection to allow for organic conversations to drive the interviews based on topics most salient to the women (Glaser & Strauss, 1967). For guidance, however, interview guides with prompts spanning a variety of topics were used during the interviews. The 2018 interview guide included key themes related to punishment, risk, perceptions of policies, and mental/physical health while the interview guide for 2019 included key themes related to perceptions of RHUs, self-esteem, coping mechanisms, relationships, advantages and disadvantages of the RHU, and perceptions of rights and privileges. For example, residents were asked “Thinking about living in the RHU, describe a story about when you felt good about yourself. What happened?” and “Discuss how your physical/mental health has changed over time while in the RHU.” Prison policy prohibits recording devices within the institutions; therefore, researchers took handwritten notes of participant responses during interviews, per traditional qualitative practice (Emerson et al., 2011). Detailed interview notes and field notes were typed after leaving the field. Due to confidentiality concerns, we did not collect women’s names during interviews—and the names from the recruitment list were not associated with the interview notes. Unfortunately, this precluded us from being able to go back into the field to have the women review and approve the final interview notes. However, following an initial review of interview notes, the research team wrote a recommendations report detailing potential changes in practices and shifts in policy to improve RHU operations for residents’ livelihoods while staying in RHUs, which was sent to prison administration. All research protocols and materials were approved by the University’s Institutional Review Board (IRB) for human subjects research with prison residents, as well as the women’s prison administration of study.

### Sample

Although the full study sample across the two time periods includes 44 women, three interviews were excluded,^3^ leaving a final sample of 41 women. Below, in Table 1, we present the demographic characteristics of the study sample excluding those two interviews. In our sample of 41 RHU women (see Table 1), most women are white or Black and are between the ages of 20 and 40 years old.

**Table 1:** Sample women demographics

Sample (*N* = 41)		
	*N*	%
**Year**		
Year 1	12	29%
Year 2	29	71%
**Unit**		
Traditional RHU	13	32%
Specialized RHU	28	68%
**Race**		
White	22	54%
Black	16	39%
Hispanic/Latine	2	5%
Multiracial	1	2%
**Age**		
20 s	14	34%
30 s	15	37%
40 s	7	17%
50 s	2	5%
60 s	2	5%
70 s	1	2%

### Coding and analysis

All typed interview notes were linked to Atlas.ti, a qualitative data management software, for coding and analysis purposes. Since a semi-grounded theory approach was used to allow for the natural emergence of themes within the data (Glaser & Strauss, 1967), two researchers adopted an open-coding, line-by-line technique to assess the topics salient to women based on those that organically arose in interviews (Charmaz, 2005). Codes created by each researcher were compared across the interview narratives and discrepancies were discussed until complete agreement was achieved. Interview narratives revealed that well-being, self-esteem, health, appearance, femininity, dignity, and decency were important topics to women although they were not directly asked about these concepts as part of the interview protocols. These topics seemed to align with the themes present in the United Nations’ standards for the treatment of prisoners. Thus, for the next round of coding, the same researchers went through the codes of interview narratives and grouped them into the following overarching categories reflecting the international standards: appearance standards, human decency standards, health standards, and well-being. This categorization process was also checked for consistency between the two researchers. For the third coding round, the researchers then went through each example of appearance standards, human decency standards, and health standards to code whether these standards were “met” or “unmet,” revealed by women using examples of what was helpful versus what was problematic in the RHU. For examples of well-being, the researchers classified well-being as “satisfactory” or “unsatisfactory,” indicative of the language women used in interviews. Similarly, the final coding round was checked for consistency between the two researchers.

After this coding process was complete, researchers connected the emergent appearance, human decency, and health standards to specific standards/rules articulated in United Nations’ documents about the treatment of incarcerated individuals. These documents include the: *United Nations Standards for the Treatment of Prisoners* (United Nations General Assembly, 2015), *Basic Principles for the Treatment of Prisoners* (United Nations General Assembly, 1990), and *United Nations Rules for the Treatment of Women Prisoners* (United Nations General Assembly, 2010). Researchers then created a cross-tabulation chart of how met and unmet United Nations’ standards/rules are connected to women residents’ sense of well-being based on interview narratives (see Table 2). This chart along with representative quotes from women residents are the basis for our findings.

**Table 2:** Residents’ well-being in RHUs (*N* = 41)

	**Unsatisfactory (*N* = 26)**	**Mixed (*N* = 4)**	**Satisfactory (*N* = 11)**
Unmet United Nations Standards			
**Appearance Unmet**	38%	50%	0%
***Clothes*** All clothing shall be clean and kept in proper condition. Underclothing shall be changed and washed as often as necessary for the maintenance of hygiene.^a^	12%	25%	0%
***Grooming*** In order that prisoners may maintain a good appearance compatible with their self-respect, facilities shall be provided for the proper care of the hair and beard, and men shall be enabled to shave regularly.^a^	31%	50%	0%
**Human Decency Unmet**	73%	100%	9%
***Nondiscriminatory Treatment*** (1) All prisoners shall be treated with the respect due to their inherent dignity and value as human beings.^b^ (2) There shall be no discrimination on the grounds of race, color, sex, language, religion, political or other opinion, national or social origin, property, birth or other status.^b^ (3) It is, however, desirable to respect the religious beliefs and cultural precepts of the group to which prisoners belong, whenever local conditions so require.^b^	46%	100%	0%
***Strip Searches*** (1) Effective measures shall be taken to ensure that women prisoners’ dignity and respect are protected during personal searches, which shall only be carried out by women staff who have been properly trained in appropriate searching methods and in accordance with established procedures.^c^ (2) Alternative screening methods, such as scans, shall be developed to replace strip searches and invasive body searches, in order to avoid the harmful psychological and possible physical impact of invasive body searches.^c^	54%	50%	9%
**Health Unmet**	85%	75%	55%
***Exercise*** Every prisoner who is not employed in outdoor work shall have at least one hour of suitable exercise in the open air daily if the weather permits.^a^	42%	75%	9%
***Hygiene*** (1) Prisoners shall be required to keep their persons clean, and to this end they shall be provided with water and with such toilet articles as are necessary for health and cleanliness.^a^ (2) The accommodation of women prisoners shall have facilities and materials required to meet women’s specific hygiene needs, including sanitary towels provided free of charge and a regular supply of water to be made available for the personal care of children and women, in particular women involved in cooking and those who are pregnant, breastfeeding or menstruating.^c^	27%	25%	9%
***Food*** Every prisoner shall be provided by the administration at the usual hours with food of nutritional value adequate for health and strength, of wholesome quality and well prepared and served.^a^	35%	50%	18%
***Medical Treatment*** Sick prisoners who require specialist treatment shall be transferred to specialized institutions or to civil hospitals. Where hospital facilities are provided in an institution, their equipment, furnishings and pharmaceutical supplies shall be proper for the medical care and treatment of sick prisoners, and there shall be a staff of suitable trained officers.^a^	31%	25%	27%
***Mental Health Treatment*** 1) At every institution there shall be available the services of at least one qualified medical officer who should have some knowledge of psychiatry. The medical services should be organized in close relationship to the general health administration of the community or nation. They shall include a psychiatric service for the diagnosis and, in proper cases, the treatment of states of mental abnormality.^a^ (2) Individualized, gender-sensitive, trauma-informed and comprehensive mental health care and rehabilitation programs shall be made available for women prisoners with mental health-care needs in prison or in non-custodial settings.^c^	46%	50%	18%
***Living Conditions*** All parts of an institution regularly used by prisoners shall be properly maintained and kept scrupulously clean at all times.^a^	23%	25%	0%
***Showers*** Adequate bathing and shower installations shall be provided so that every prisoner may be enabled and required to have a bath or shower, at a temperature suitable to the climate, as frequently as necessary for general hygiene according to season and geographical region, but at least once a week in a temperate climate.^a^	42%	75%	18%

^a^ United Nations Standards for the Treatment of Prisoners

^b^ Basic Principles for the Treatment of Prisoners

^c^ United Nations Rules for the Treatment of Women Prisoners

## Findings

Women describe the bleak conditions associated with living in an RHU such as refusal of hygiene items, poor medical care, constant strip searching, inadequate bathing time, and more—all indicative of feeling like they were being treated as animals. Oftentimes, these conditions violate the standards set forth by the United Nations regarding the treatment of incarcerated individuals. In particular, we identified 16 specific United Nations standards—grouped into the larger themes of appearance, human decency, and health—that the narratives from women living in the RHU suggest are not being met. Overall, 29% of the women identified two appearance standards that are unmet, 57% of the women identified five human decency standards that are unmet, and 76% of the women identified nine health standards that are unmet. Women’s perceptions of these prison conditions can then be linked to their sense of well-being. As shown in Table 2, we connect 16 specific United Nations standards related to appearance, human decency, and health with women’s sense of well-being.

Consistent with prior research (Arrigo & Bullock, 2008; Cloyes et al., 2006; Grassian, 2006; Haney, 2003; Haney & Lynch, 1997; Reiter, 2016; Reiter et al., 2020; Strong et al., 2020, Browne et al., 2011), many of the women in our sample (63%) state that living in an RHU negatively impacts their physical health and/or mental state, which we are deeming “well-being.” For example, Chelsea^4^ describes the RHU as “traumatizing both mentally and emotionally,” even stating, “I don’t think it’s beneficial at all.” Susan remarks that “Solitary is hard on my mind…If they keep me any longer, I’m going to lose my mind. It’s getting worse over time.” Other women describe feeling “trapped,” “lost,” and “forgotten” in the RHU. Multiple women admit they either have no self-esteem or exceedingly low self-esteem while living in the RHU.

The women whose well-being is negatively impacted by the RHU are much more likely to perceive that appearance (38%), human decency (73%), and health (85%) standards are inadequately addressed in comparison to women who say their well-being is satisfactory. Not all women feel their well-being suffers while in the RHU, as exemplified by Jackie and Tasha who echo each other saying, “I’m in good health,” and “I’m in good mental health.” They are among the 27% of women who feel their well-being is satisfactory in the RHU; for these women, none feel appearance standards are unmet, only 9% feel human decency standards are unmet, and 55% feel health standards are unmet. The final group of women (10%) describe their well-being as a mix between satisfactory and unsatisfactory. As an example, Latishia says she has high self-esteem, but later says she feels “all the way disconnected” in the RHU. This group of women heavily perceive that appearance (50%), human decency (100%), and health (75%) standards are not being properly adhered to while in the RHU. To further explore the connection between neglected standards and resident well-being, in the following sections we detail the specific unmet appearance, human decency, and health standards outlined by the United Nations that seemingly drive residents’ sense of well-being.

### Unmet appearance standards

Maintaining a decent appearance while confined is often a requirement of prison residents and an unkept appearance is grounds for a misconduct charge. For women, it could be argued that this standard is even higher, which is upheld not only by staff, but by the women themselves. The women we interviewed describe how limited access to mirrors and razors and problematic clothing make it difficult for them to maintain their appearance (much less achieve any sense of beauty). The blocked usage of mirrors and razors directly contradicts the United Nation’s provision that incarcerated individuals be provided with the appropriate facilities to “maintain a good appearance compatible with their self-respect.” This is an individual achievement for residents, but the tools to accomplishment such a feeling are only available through the actions of RHU staff.

To garner a sense of self-worth through one’s appearance standards being met, mirrors are often a mechanism to aid in the process. For instance, as Kiki states, “A mirror would help with my self-esteem.” Similarly, Holly says you “need a mirror in your room” because “if you don’t see yourself, your self-esteem gets low.” And, according to Sara, “you go crazy if you can’t see yourself.” For women, the lack of access to a mirror to view themselves physically impacts their self-esteem due to their inability to maintain their desired appearance. To combat this, women attempt to find a reflection in other ways, such as in a toilet bowl like Holly or in a window like Sue and Rebecca. Rebecca explains that the administrative solution to the mirror problem in the RHU was performative at best because staff put a couple of mirrors in the shower, but “that’s dumb because the hot water just fogs it up.” She thinks that RHU staff installed the mirrors to say they accomplished what was asked of them, but that it is “pointless” when mirrors cannot be successfully used in the shower.

Women in RHUs also have limited access to razors due to time limits in the shower and razors being a privilege that can be revoked anytime. Women have the privilege of using a razor during shower time (Mondays, Wednesdays, and Fridays) if they sign up for one in the morning, but as Rebecca highlights, “shaving is a whole other time commitment in itself and there are only 10 min” allowed in the shower altogether. This made women feel like they have to choose between getting clean or shaving. However, making this choice produces a recurring sentiment among women like Ramona who indicates, “Not shaving makes me feel like an animal.” This pain is heightened for one of the transgender women in the RHU because her beard keeps growing in, and it is troubling for her. She says, “I look like a crazy old man and I hate it. It makes me want to smash my head off the floor. I feel really gross.” These women highlight how a simple tool that most people in the general public take for granted can make women feel completely unlike themselves because they do not have the ability to shave.

A few women also discuss issues they have with clothing, despite the United Nation’s mandate that “all clothing shall be clean and kept in proper condition.” Holly complains that “you don’t get a new jumper just because you get a shower” so “you have to shower and put your dirty jumper back on.” Sara and Julie have issues with the cleanliness of jumpsuits as well, but their complaints were in relation to what happens when they get their period and bleed through their clothes. Sara explained if they bleed onto their clothes:

You have to wash out your underwear and your oranges [jumpsuit] in the sink. They won’t take them to the laundry with blood on them…you can’t get clean ones if you don’t turn in a pair…it’s gross.

Simply obtaining access to clean clothes becomes difficult in the RHU because there is no in-unit laundry; the location of the washers and dryers, along with the resident worker role associated with doing laundry make convenient and frequent access to clean jumpsuits irregular. The experiences of the women illustrated above reveal the simplicity of having access to items that make women in the RHU feel like they can obtain an appearance they are content with, let alone achieve a notion of femininity by being well-kept.

### Unmet human decency standards

The absence of basic human decency is a major complaint for women who believe their well-being is suffering in the RHU. Women regularly report feeling dehumanized, degraded, and humiliated and are highly aware of their lack of bodily autonomy and privacy. Rebecca explicitly states, “They *dehumanize* us here,” which joins a common refrain from the women that they are treated like “animals.” For instance, Susan admits, “I’m treated like I’m in a zoo,” highlighting the comparison between her treatment and that of zoo animals. This is echoed by Ramona when she says, “I am tired of being treated like an animal.” Rachel further describes that, “They put us in cages, take us out on leashes like we are dogs.” Several other women compare their lives to animals and their daily routines to being trapped in a cage. This led to women indicating they did not feel human while confined in the RHU, such as Holly who says, “In here I feel less than a human, I don’t know who I am anymore.” More specifically, women in the RHU describe how they feel degraded and humiliated by officers who refuse to respect religious rituals, engage in name-calling, deliberately mispronounce women’s names, mis-gender transgender residents, and provoke and harass the women. Rachel sums up the RHU experience by saying it is “Humiliating. They take away your pride and dignity.” These women’s experiences highlight the violations in the United Nation’s standards committed by RHU staff within a larger correctional institution, even though the standards state incarcerated individuals should be treated with respect, dignity, and in a nondiscriminatory way.

The constant strip searches and lack of privacy in RHUs is also painful for women because they lose autonomy over their bodies. Unlike the general prison population, it is RHU policy for women in the RHU to be strip-searched every time they leave their cell. Rachel points out that, “We have to take all our clothes off and some of the staff won’t hold up the shirt so everyone can see you,” which as she puts it, is “humiliating.” Julie echoes this and says she “doesn’t like to do strip searches” because “it’s embarrassing.” Rebecca voices her concerns as it relates to privacy, “We go to shower—we’re stripped down; we go to rec—we’re stripped down; and when we go to leave the cell—we’re stripped down.” She did not understand why the prison policy is so adamant on finding contraband on one’s body, when “we don’t have nothing” and that staff go to the lengths of completely dehumanizing people in the process of searching for it. Both Ruby and Lydia believe women in the RHU should have more privacy because “everyone can see everything.” These stories indicate that effective measures have not been taken “to ensure that women residents’ dignity and respect are protected during personal searches,” nor have alternative screening methods been developed “to replace strip searches and invasive body searches, in order to avoid the harmful psychological and possible physical impact of invasive body searches” on women residents (United Nations General Assembly, 2010, p. 12).

### Unmet health standards

The neglect of health standards is a common issue for women RHU residents, even among those who perceive their well-being as satisfactory. Some neglected health standards include: lack of exercise time, lack of shower time, poor medical care, poor mental health care, inadequate hygiene items, inadequate quality and quantity of food, and the unsatisfactory quality of the physical environment of the RHU. These are frequent United Nations’ health standards the women cite as not being met appropriately. Some of these unmet health standards result from formal RHU policy, but others are unmet due to correctional officer discretion and lack of resources. For example, the lack of exercise time and shower time are largely due to RHU policies that dictate women can only be given five hours of exercise and three showers per week. Many women point out that this is a distinct disadvantage of living in an RHU and Rebecca elaborates that “all weekend they get nothing—no rec, shower, or anything. It causes people to break down.” Confirmed by multiple women, Kiki also reveals that, “They give you 10 min in the shower maximum.” However, some women describe how correctional officer discretion contributes to their lack of exercise and shower time. Being “burned” [denied] or “gypped” of exercise and/or shower time is a common experience women describe; it occurs when correctional officers purposefully deny women the opportunity to shower or go to yard or cut the designated time for those activities short.

In contrast, it may be a scarcity of resources that contributes to the poor medical care and mental health care the women in the RHU report. Juanita explains:

There are not enough officers here to get you to your medical appointments all the time, so you can be severely ill and not get the care you need.

While Juanita finds fault with the ability of officers to get women to their medical appointments, other women complain about how they are either given the wrong medication or not given medication at all. For example, Kiki has been without her medication “for months” and Beth feels “defeated” because medical keeps giving her the wrong medications. With regards to mental health care, women in the RHU—even those in specialized RHUs specifically for women with diagnosed mental health conditions—feel they are not getting the help they need and that the counselors/psychologists “don’t help.” Several women complain that because there are so few counselors/psychologists, they cannot get one-on-one counseling but the state’s DOC has a policy indicating that individuals housed in RHUs are supposed to receive contact with mental health counselors at least once every 30 days or once every 14 days if in a specialized RHU. Ebony says she is “supposed to be able to talk to the psychologists in person, but they are not always able to see me when I put in a request.” Brianna further elaborates that even when they respond to her request to talk to a counselor/psychologist “it’s not for very long and not usually confidential—it’s at the cell door” even though the state’s DOC policy requires “out of cell” sessions. This was a common complaint; other women in the RHU are often privy to the intimate details women share with the counselors/psychologists because the counseling takes place at the cell door or in group therapy, meaning other women residents are present, or within ear-shot, to be able to hear personal conversations about mental health concerns taking place. According to Deja:

We get to talk to the psychiatrist, but they talk at the door to our cells and it’s not private. I never get to address my feelings. Maybe if I could talk out some of my feelings in private maybe I wouldn’t self-harm. I’m not going to talk in front of other inmates…they would use my feelings against me.

This sentiment is echoed by several other women who feel unwilling to disclose information to counselors/psychologists for fear the other women and correctional officers will hear and use the information against them.

Policies for RHUs, scarcity of resources, and correctional officer discretion all seem to play a role in women’s perceptions that the hygiene products and food in the RHU are inadequate. Per RHU policy, women are given basic hygiene products (e.g., toothbrush, toothpaste, shampoo, bar soap, sanitary pads), but if they want additional items (e.g., conditioner, tampons), they must buy them from commissary received once weekly. Women in our study indicated that they could ask for additional items such as tampons or pads if they run out, but depending on the correctional officer, they may or may not receive more. Across the women, there was no consensus on the policy of how many tampons or pads could be received per day—it ranged from two, to six, to eight, to eighteen. And, there is no required number of tampons or pads allotted to individuals according to the state’s DOC policy. Lydia offered an explanation as to why there might be confusion or why practices may have changed regarding allowances of menstrual products when she says, “Once we got a female superintendent, things got better.” However, for women with no money for commissary earned from prison labor or received by family/friends, they have to make do with the basic items provided by the prison or asking correctional officers and other residents for items. This meant women had to make do with what they had until they were able to acquire more. For instance, women frequently run out of items such as toilet paper, deodorant, toothpaste, and period products and it is up to correctional officers’ discretion whether to provide more once women use up their allocation required by policy; in practice, allotments of items often vary from the perceived policy due to officer discretion.

Food is also viewed as inadequate as Rebecca states, “The food is awful, you lose weight, and you’re barely fed.” The perceived quality and quantity of the food provided by the RHU is insufficient according to many women, along with the timing of the meals being delivered. In the RHU, they serve dinner at 4 p.m. and Ruby describes why this is a problem, “On Sunday we got a grilled cheese for dinner. That was all. We ate the grilled cheese at 4 p.m. and then nothing until the next morning.” Therefore, if women do not have the means to purchase additional food from commissary or manage to keep and successfully hide food from previous meals such as fruit or chips, they often go without food for up to 14 h a day—and there is no remedy written within the state’s DOC policy for this lack of food without means to purchase more. Compounding these inadequacies with the food, multiple women indicate that similar to what happens with yard and showers, some correctional officers will “burn”^5^ women for food. According to Carla, correctional officers “won’t give you food if you’re not at the door when they yell trays.” In this way, receiving the necessary items to stay healthy, to stay alive, and to stay well are bounded by structural barriers that make them difficult to secure.

Lastly, some women perceive the physical environment of the RHU to be unhygienic and unclean. Perhaps due to scarce resources, cells and showers are not cleaned frequently enough and women describe them as “filthy,” containing “mold,” and as “not sanitary.” While residents are considered to be inside workers whose duty it is to clean these spaces, there is often not enough allotted time or products to do so on a regular basis or resources to fix ongoing issues outside of residents’’ control (e.g., mold). Additionally, it is fairly common for residents to throw feces and urine at correctional officers in a show of grievance or as a result of untreated mental health issues. Daniela describes being afraid of getting sick because some women “spread feces and urine all over the place.” This perception that the women are creating some of these conditions themselves causes correctional officers to use their discretion in a way that delays the clean-up process often making it harder to rinse off and then disinfect at a later time. For example, Ramona discusses how her cell toilet flooded and correctional officers left her in the cell without a flushable toilet for days because she “did it to herself” so she needed “to live with it.” Susan has a parallel story discussing when her toilet flooded, correctional officers left her “in the dirty cell all day long. They are supposed to clean the cells, but they don’t.” In terms of cleanliness, there appears to be an apparent disconnect between RHU staff and residents as to whose role it is to maintain a clean unit. What all of these examples illustrate is how women living in RHUs feel basic health standards are not being met and this is problematic for their overall well-being.

## Discussion

Of the 41 women in our sample, most express their well-being while living in an RHU is poor, at least at times, when appearance, human decency, and health standards are unmet. In conjunction, of all of these needs going unmet, a handful of women compare their treatment to that of zoo animals. Women reference not being able to keep up their appearance through shaving or with appropriate shower time, being caged and mistreated by officers’ verbal abuse, being discriminated against by officers for holding marginalized identities, being humiliated and having their privacy violated during strip searches, not being able to access basic services or only being able to do so through a door and being given little to no food and/or substandard, innutritious food. Sub-par, and arguably inhumane, treatment from correctional officers, whether of their own volition or according to institutional policies, was viewed by women as likened to how people treat caged animals. While the state’s DOC policies use mostly non-specific language—or the rules are non-existent—regarding some of the concerns expressed by women residents in this study, the treatment described by them reflects that their day-to-day lives within solitary confinement units are outside the acceptable treatment of prison residents on a global scale.

Since women who are incarcerated are at an increased likelihood to have a SMHD (LaChance, 2018) and residents with SMHDs are subjected to solitary confinement more often (Houser & Belenko, 2015; Wright et al., 2007), it is unfortunately not surprising that a disproportionate number of women experience solitary confinement while incarcerated. Comparatively, women are diagnosed with mental health disorders at five times the rate of men who are incarcerated (Drapalski et al., 2009; Hills et al., 2004; James & Glaze, 2006). However, the breadth of research spanning from Sykes (1958) to Strong et al. (2020) studies detail the physical and mental health issues of residents worsen during and following a stint in solitary confinement. In addition, access to mental health treatment in women’s prisons is inadequate and, sometimes, even nonexistent (The Sentencing Project, 2020). This is often because funding for treatment is usually allotted to the large number of incarcerated men in U.S. prisons (Magaletta et al., 2009).

This body of literature highlights why residents’ experiences within these harsh environments are often referred to as “prisons within prisons;” they are an even more punishing place. This is because treatment within solitary confinement produces additional harms to residents, like those detailed above for the women in our study, often making residents feel “like an animal.” In this way, the inherently deprived environment of prisons does not allow for mental and physical health problems to be addressed or rehabilitated (Acoca & Austin, 1996; Enders et al., 2005) because they are instead exacerbated while in solitary confinement. Our study expands on previous knowledge of the negative impacts of solitary confinement by linking women residents’ well-being to specific harms experienced in solitary confinement. Currently, Western et al. (2021) are the only other researchers who have examined how specific conditions of solitary confinement are linked to residents’ well-being, but their study occurred in a men’s prison. In the future, alternatives to and improvements in solitary confinement are necessary. In a study of prison administrators, Mears et al. (2021) found that when asked about alternatives to solitary confinement, “many of them emphasized that the main “alternative” would be to improve overall prison system operations by increasing the number and quality of staffing, offering more rehabilitative programming, creating productive routines to keep inmates busy, and incorporating more evidence-based interventions and practices” (p. 645). Our study highlights focal points that if improved, could positively impact the lives of women residents within solitary confinement.

### Recommendations

Our study illuminated that women confined in solitary confinement have heightened needs for resources to address mental health, appearance, individual identity expression, and bodily autonomy. Further, we explicitly highlight that perhaps like this one women’s prison, American prisons are not upholding the rights of residents according to the United Nations’ standards. It is not normative for them to do so as they have not committed to following the United Nations’ international standards because prisons in the United States are decentralized, meaning states create policies applicable only to their DOC and the prisons within it. We offer recommendations for better adherence to the United Nations’ international standards for the treatment of prisoners. Additionally, recommendations were also iterated to prison administration following data collection. In the future, practitioners, politicians, and scholars have the opportunity and the obligation to promote the implementation and evaluation of more humane practices and policies for the treatment of residents by correctional officers and administration (such as those laid out in the United Nations General Assembly, 1990, 2010, 2015) and seek to ensure that all prison residents are able to experience a prison environment that allows them the opportunity to be rehabilitated, not reharmed. To do so, global alternatives to solitary confinement such as increased programming, therapeutic housing units (e.g., “step down” programs to reintegrate residents back into general population, mental health units) (Walsh et al., 2020), may be worth evaluating for increased implementation within the United States.

In terms of resident treatment by officers, correctional administration should seek to incorporate the United Nations’ basic standards for treatment via collaboration with members of the United Nations to better understand the boundaries of the guidelines, share global ideas of other prison strategies that work, and offer technical assistance to ensure implementation is effective. For example, this women’s prison administration could seek to learn what technology exists, where to purchase it, and how to use it for alternative screening methods for strip search purposes rather than physically touching residents’ bodies. At a higher level, the state’s DOC policies could be expanded upon to become more specific in regards to the placement of mirrors within housing units, additional allocations of food beyond required meals and commissary purchases, minimum allotment of period products within a given time frame, and protocols for medication administration accuracy. Beyond the simplest recommendation that the U.S. carceral system attempt to follow the United Nations’ standards for the treatment of prisoners, there are more specific practical changes that could be made to promote a more livable experience for women living in this case study prison’s RHUs.

Overall, the accessibility of wellness products and resources can be improved. For one, making all personal (e.g., soap, shampoo, toothpaste) and period products (i.e., tampons and pads) free for residents as they are required to retain one’s cleanliness and remain compliant with the prison appearance code should be considered. If a limit or restriction on free items is necessary to assure items are not misused or pose a safety risk, then ensuring practical enforcement of it is warranted rather than as a punishment for misbehavior. Further, shower times of 10 min within solitary confinement units like RHUs should be lengthened to 15 min when “razor privileges” are in effect to allow women adequate time to clean themselves and shave. Fifteen minutes is perhaps a reasonable amount of time for women to both clean and shave, without unduly burdening staff or drastically changing the day’s schedule. To allow residents to see whether or not they are compliant with the prison appearance code according to their own views, state and federal prisons could contract out a paid opportunity to design a reflective surface similar to a mirror that is not a potential safety hazard for residents to use. Additionally, prison administration could hire third party cleaning services from outside the institution to ensure compliance with sanitary standards as verified by the Bureau of Prisons’ annual operations inspections, and that may promote a greater sense of satisfaction among residents on the cleanliness of facilities; they may be able to work in conjunction with or provide training to inside prison workers whose assigned jobs are to clean the prison facilities already. Lastly, academics and advocates have encouraged it for years, but residents should be fed better using foreseeable food waste from companies, restaurants, or co-ops willing to partner with correctional facilities to provide or make available for purchase unused food. These are simple and specific ways to change the physical environments and daily routines of prison residents within this one women’s prison but especially those within solitary confinement units that do not require a complete overhaul of the entire carceral system to improve the well-being of women residents.

Lastly, there should be an attempt to develop and infuse an interdisciplinary educational model on resident well-being into correctional officer training curriculum that involves the actual experiences and feedback of residents themselves—on top of the additional training for officers assigned to work in solitary confinement given the additional security measures it entails. Such a model could be created by prison residents, academic scholars, correctional practitioners, rights advocates/activists, attorneys, and non-government organizations to build learning tools, activities, and courses tailored to the improving the process of correctional officers’ interpreting prison policies and practically applying discretion on the job. This model could lay the foundation for at least the bare minimum information on topics that may not readily be offered outside of the traditional job duties of ensuring safety and control, such as evidence-based corrective care, religious importance, benefits of exercise, challenges of disabilities, biological understanding of bodily functions, and gender-specific needs. Using the United Nations’ standards as the basic threshold for the treatment of residents, teaching officers what else would be required for residents to achieve satisfactory well-being while incarcerated is valuable for multiple reasons. If residents perceive they are being treated well and they feel good (mentally and physically), this could reduce requests for items and services and residents may be less likely to engage in acts of deviance in order to garner the items and services they need (see Ingel et al., 2021b for further discussion). This would not only make the carceral experience of residents better, but also reduce the stress and strain of correctional officers tasked with overseeing residents especially in solitary confinement where restrictions are more common.

### Limitations

As for limitations, our sample is of adequate size with 41 participants and is somewhat diverse, but yielding more participation from a greater variety of women residents with multiple intersecting identities would make our results richer. For example, we were not able to fully capture the experiences of women who speak Spanish as no one on our research team was bilingual. Due to the relatively small number of women across demographic categories and to ensure the anonymity and confidentiality of the information shared during the study, no further analyses of women residents’ demographics (e.g., race, age) or intersecting identities were conducted to try to explore varying themes of women with potentially nuanced prison experiences. While we did not compare across race, ethnicity, or age of the women in the study, we recognize the importance of doing so in that Black women are twice as likely to be charged with verbal infractions and subsequently sentenced to solitary confinement than white women (Pullen-Blasnik et al., 2021). Future research on the treatment of women in prison should examine the varying experiences of women of color, women with disabilities, Muslim women, women born outside the U.S., women that do not speak English, and more. This is especially true considering solitary confinement is often used to house populations perceived to be at increased risk of harm or need of protection such as residents with disabilities, pregnant residents, or residents who identify as LGBTQIA + (Fettig, 2020).

In addition, the information shared by women RHU residents on their experiences in prison specific to appearance, human decency, and health were not topics directly asked about in the study protocol given that we used a semi-grounded theoretical approach to interviews. However, we found the points that women residents made still clearly highlighted problems with meeting the United Nations’ standards for the treatment of prisoners, especially those in the RHU. While not explicitly an aim of the original study, the salience of the experiences of women RHU residents was pertinent enough for them to organically discuss their unmet needs in interviews and reveal appearance, human decency, and health themes which mapped onto the United Nation’s standards well.

### Future research

While it would be particularly challenging, attempting to examine whether or not there is global adherence to the United Nations’ international standards for the treatment of prisoners is necessary, but especially in the U.S. where the prison population is mounting to over two million people. It may also be useful to compare prisons and jails to determine whether they significantly vary in terms of procedures that align with the United Nations’ international standards. Doing so would illuminate the goals, practices, and standards being carried out and reveal what standards need to be further addressed. Similarly, future research could seek to explore effective methods to enforce the United Nations’ standards around the world, especially since incarceration is still widely used and will not likely be abolished in the near future.

Furthermore, comparing across countries with different adherence practices and enforcement mechanisms is valuable for additional examinations of residents’ well-being with the potential for recidivism reductions. In doing so, research could attempt to shed light on whether better treatment of prison residents impacts their likelihood to recidivate. Studying these new avenues of research pose great opportunities for increasing our breadth and depth of knowledge on a geographical level as well as institutional level adherence to prisoner rights. In turn, those results have the potential to be translated into positive changes within prisons/jails for the improved well-being of the lives of those imprisoned. The humane treatment of people who are confined and incapacitated is morally right, but people are also entitled to such treatment as a basic human right as purported by the United Nations.

## Data Availability

The dataset analyzed during the current study is not publicly available due to privacy, confidentiality, and anonymity of study participants.

## Footnotes

1 All of the researchers who participated in this trip were English-only speakers. However, this did not impede our research as we did not encounter any women who did not speak at least a little English.

2 Psychiatric cages are locked spaces resembling cages made of metal bars and a wicket—a hole/slot in the door for the placement of hands or transference of items—that can fit one person inside with a chair for sitting or room for standing and they are typically located in rooms where programming/services occur.

3 One interview was stopped by a researcher due to competency concerns. A second interview was stopped early at the request of the resident for an unknown reason. The third interview was excluded because it did not meet our analysis criteria.

4 All participant names mentioned are pseudonyms.

5 “Burn” in this context means to refuse to give residents something, such as shower time, access to yard, or food.
